# The Putative HORMA Domain Protein Atg101 Dimerizes and Is Required for Starvation-Induced and Selective Autophagy in *Drosophila*

**DOI:** 10.1155/2014/470482

**Published:** 2014-05-08

**Authors:** Krisztina Hegedűs, Péter Nagy, Zoltán Gáspári, Gábor Juhász

**Affiliations:** ^1^Department of Anatomy, Cell and Developmental Biology, Eötvös Loránd University, Budapest 1117, Hungary; ^2^Faculty of Information Technology and Bionics, Pázmány Péter Catholic University, Budapest 1083, Hungary

## Abstract

The large-scale turnover of intracellular material including organelles is achieved by autophagy-mediated degradation in lysosomes. Initiation of autophagy is controlled by a protein kinase complex consisting of an Atg1-family kinase, Atg13, FIP200/Atg17, and the metazoan-specific subunit Atg101. Here we show that loss of Atg101 impairs both starvation-induced and basal autophagy in *Drosophila*. This leads to accumulation of protein aggregates containing the selective autophagy cargo ref(2)P/p62. Mapping experiments suggest that Atg101 binds to the N-terminal HORMA domain of Atg13 and may also interact with two unstructured regions of Atg1. Another HORMA domain-containing protein, Mad2, forms a conformational homodimer. We show that *Drosophila* Atg101 also dimerizes, and it is predicted to fold into a HORMA domain. Atg101 interacts with ref(2)P as well, similar to Atg13, Atg8a, Atg16, Atg18, Keap1, and RagC, a known regulator of Tor kinase which coordinates cell growth and autophagy. These results raise the possibility that the interactions and dimerization of the putative HORMA domain protein Atg101 play critical roles in starvation-induced autophagy and proteostasis, by promoting the formation of protein aggregate-containing autophagosomes.

## 1. Introduction

Autophagy ensures the lysosome-mediated degradation and recycling of cytoplasmic components including organelles. During the main pathway, a phagophore cistern (also called an isolation membrane) forms and captures cargo destined for breakdown into a double-membrane autophagosome. This vesicle then fuses with a late endosome or lysosome containing acidic hydrolases. Assembly of the phagophore is achieved by the action of Atg proteins.* Atg* genes were originally discovered in yeast, and the majority of them have clear orthologs in higher eukaryotes including animals [1, 2].

Initiation of autophagy usually begins with the activation of an Atg1 protein kinase complex in animal cells, which contains the serine/threonine kinase Atg1 (its orthologs are called UNC51 in worms and ULK1 and 2 in mammals), Atg13, FIP200/Atg17, and the metazoan-specific subunit Atg101 [3]. This complex directly binds to Tor (target of rapamycin) kinase, which is active when bound to digesting lysosomes and promotes cell growth and inhibits autophagy by phosphorylating Atg1 [4, 5]. Autophagy-inducing stimuli such as starvation rapidly lead to inactivation of Tor and induction of Atg1-dependent autophagy [3, 6]. This results in the removal of inhibitory phosphogroups from Atg1 by poorly characterized phosphatases, which may potentially include PP2A [7]. Atg1 then undergoes autophosphorylation on residues separate from those phosphorylated by Tor and also phosphorylates downstream targets including Atg13 [3, 8, 9]. Overexpression of Atg1 strongly promotes autophagy in* Drosopohila*, while expression of a kinase dead form suppresses starvation-induced autophagy [10].

It is largely unknown how the Atg1 kinase complex transmits its autophagy-inducing signal to downstream components, which include an autophagy-specific lipid kinase complex, phospholipid effectors such as Atg18, the transmembrane protein Atg9, and two protein conjugation systems that include the ubiquitin-like Atg8 family proteins [1, 3]. Atg8 is conjugated to phosphatidylethanolamine, and thus it is bound to phagophore and autophagosome membranes [11, 12]. Atg8, and its mammalian homologs such as LC3, binds to cargo receptors including p62 to mediate the selective autophagic breakdown of ubiquitinated protein aggregates [13, 14]. Ref(2)P, the fly homolog of p62, is required for the formation of these protein aggregates in* Drosopohila* [15].

FIP200/Atg17 is thought to act as a scaffold protein in the Atg1 kinase complex, whereas the role of the metazoan-specific subunit Atg101 is poorly characterized [3]. Atg101 (also known as C12orf44) is a subunit of the Atg1/ULK complex in human cells, and shRNA depletion of* Atg101* impairs autophagy in mammalian cells [16, 17]. Moreover, the* Atg101* ortholog* epg-9* has recently been shown to be required for degradation of P granule aggregates in worm embryos as well [18]. As the precise role of Atg101 in autophagy is unclear, we decided to analyze its function in* Drosopohila*.

## 2. Materials and Methods

### 2.1. *Drosopohila* Genetics

Flies were reared on standard yeast-cornmeal-agar medium (fed), and mid-L3 stage larvae were transferred to a 20% sucrose solution for 3 h for starvation experiments. Fat body cell clones expressing RNAi constructs* Atg101[KK106176]* and* Atg101[HMS01349] *were generated spontaneously, as described in detail previously [19–22].

### 2.2. Histology

Dissected larval carcasses were incubated in a solution of LysoTracker Red (LTR) and DAPI as before [19–23]. Fixed samples were processed for indirect immunofluorescence using rat anti-Atg8a [9] and rabbit anti-ref(2)P [22] and imaged as described in detail previously [19, 20, 22]. Dissected fat body lobes containing* Atg101 *RNAi clones were attached to poly-L-lysine-coated coverslips in PBS, photographed live to record the position of GFP-positive cells, fixed and embedded into Durcupan (Fluka), and then sectioned and analyzed by transmission electron microscopy as described [19, 21]. Statistical analysis was carried out as described [19, 20].* N* refers to the number of animals, and multiple cells were evaluated from each animal.

### 2.3. RT-PCR

Total RNA was isolated from starved L3 stage larvae of the genotypes **w*[1118]* (used as wild type),* Atg101[KK106176]/+, Act-Gal4/+, *and* Atg101[HMS01349]/Act-Gal4 *using PureLink RNA Mini Kit (Invitrogen), followed by preparation of cDNAs using RevertAid First Strand cDNA Synthesis Kit (Thermo) and RNase free DNase I (Sigma). PCR reactions on cDNA samples were performed with the following primers: TACTCCTCCCGACACAAAGC, CTGGGTCATCTTCTCACGGT for* Actin5C* (23 amplification cycles), and ATGAACGCGCGTTCGCAG, TCACATTGCGAGCGTTTCCT for* Atg101* (25 amplification cycles). Parallel reactions were performed without adding reverse transcriptase to control for DNA contamination. As expected, no PCR products were obtained in these experiments.

### 2.4. Cell Culture and Immunoprecipitations

*Drosopohila* D.Mel-2 cells (Invitrogen) were used for transfection and immunoprecipitation experiments as recently described elsewhere [9, 19, 20]. The following constructs were used in coimmunoprecipitation experiments: an N-terminally truncated 3xFLAG-ref(2)P lacking the PB1 domain mediating self-aggregation, 3xHA-Keap1 [20], 3xHA-Atg18 [24], 3xHA-Atg101, full-length 3xHA-Atg13 and its N-terminal, middle, and C-terminal fragments [9], and kinase dead myc-Atg1 [8]. 3xHA-Atg8a, 3xHA-Atg16, 3xHA-RagC, 3xFLAG-Atg101, and Atg101-3xFLAG were generated by PCR amplifying the full-length coding sequences from a cDNA sample and cloning these into appropriate UAS vectors, respectively. GST coding sequence was PCR amplified and cloned into a UAS-3xHA vector downstream of the HA tag. 3xHA-GST-Atg1 constructs were generated by PCR amplifying and cloning the appropriate Atg1 fragments downstream of GST.

### 2.5. Bioinformatics

Sequence analysis and motif search were performed using the NCBI BLAST service as well as the Pfam (version 27.0) and Prosite (release 20.97) databases and their associated search tools. Alignments of orthologous sequences were extracted from OMA groups as cross-linked to the corresponding SwissProt entries [25]. For structure prediction, the I-TASSER and Phyre2 servers were used [26, 27]. Pairwise and multiple structural alignments were done with DaliLite and MAMMOTH-Mult, respectively [28, 29]. The ANCHOR and IUPred servers were used to predict unstructured regions and potential binding sites within these regions in fly Atg1, Atg13, and Atg101 [30, 31]. Structure visualization was performed using UCSF Chimera [32] and the Prosite HORMA logo was reproduced with WebLogo [33]. The alignment of human, fly, and worm Atg101 was generated using CLUSTALW and colored with TEXSHADE at the Biology Workbench server [34].

## 3. Results and Discussion

### 3.1. *Drosopohila* Atg101 Is Required for Starvation-Induced Autophagy

**Figure 1:**
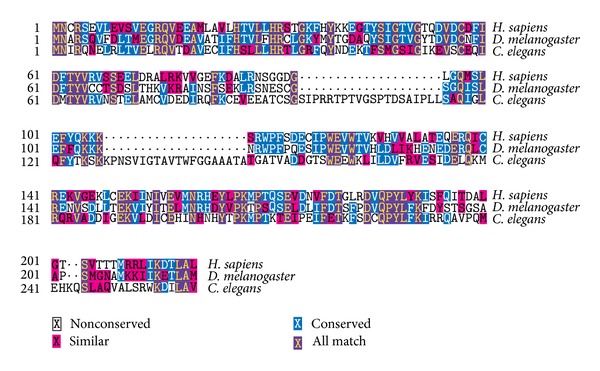
Multiple sequence alignments of human, fly, and worm Atg101 proteins.

**Figure 2:**
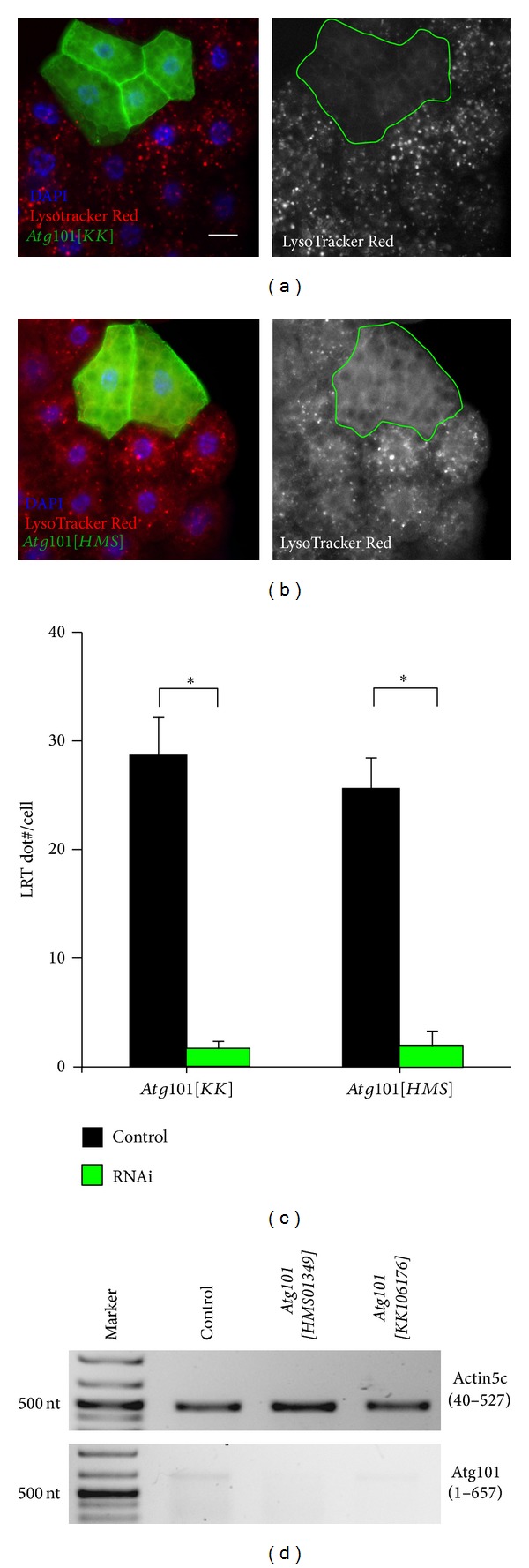
RNAi depletion of* Atg101* by expression of a long hairpin (a) or a microRNA-based silencing construct (b) in clones of cells marked by membrane-bound mCD8-GFP prevents starvation-induced punctate LysoTracker Red (LTR) staining, compared to surrounding control fat body cells of third instar larvae. (c) Quantification of data shown in (a) and (b), *N* = 5, *P* < 0.001, two-tailed, two-sample Student's* t*-tests. (d) Reverse Transcriptase-PCR analysis reveals that* Atg101* mRNA expression is strongly reduced upon expression of* Atg101[KK],* and it is undetectable in* Atg101[HMS]* expressing animals compared to wild type L3 stage larvae. Note that the expression level of* Atg101 *is much lower than that of* Actin5C*, a commonly used control in such experiments. Numbers in parentheses indicate the amplified region of the coding sequence of these genes. Scalebar equals 20 *μ*m for microscopic images.

**Figure 3:**
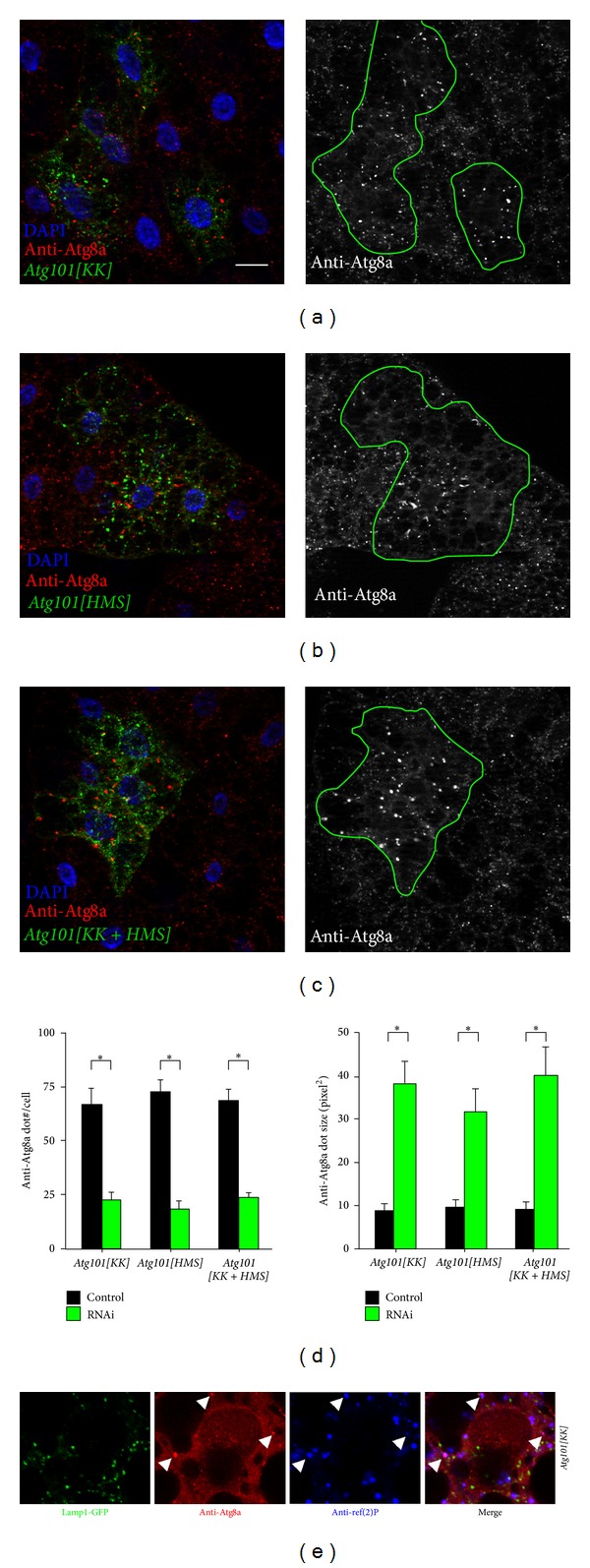
Knockdown of* Atg101 *using separate RNAi lines ((a), (b)), or the combination of both (c), impairs the formation of Atg8a-positive autophagosomes upon starvation. Note that fewer but bigger Atg8a puncta are seen in Lamp1-GFP-marked RNAi cells than in neighboring control fat body cells. (d) Quantification of data shown in (a) to (c), *N* = 5, *P* < 0.001, two-tailed, two-sample Student's* t*-tests. (e) Overlapping Atg8a and ref(2)P structures are highlighted by arrowheads in* Atg101* depleted cells. Scalebar equals 20 *μ*m.

**Figure 4:**
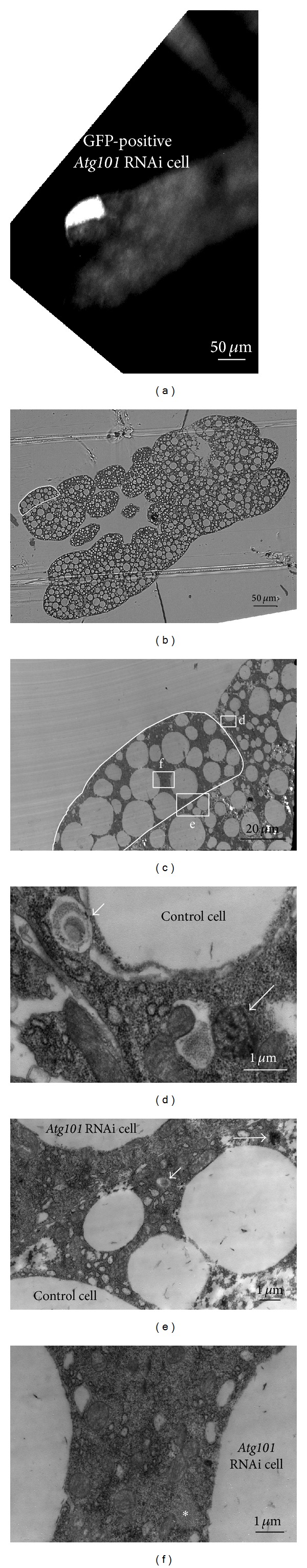
Ultrastructural analysis of* Atg101[KK+HMS] *RNAi cells. A fat body lobe containing a single GFP-marked RNAi cell (a) was embedded in plastic, and the same cell is highlighted in semithin (b) and low-magnification ultrastructural images (c). High magnification images show autophagosomes (short arrows) and autolysosomes (long arrows) in control cells ((d), and also in (e), bottom). The generation of such autophagic structures is inhibited in* Atg101 *RNAi cells ((e), top, and (f)). Asterisk marks a potential cytoplasmic protein aggregate, which can be recognized by its homogenous appearance and exclusion of organelles and ribosomes.

BLAST searches reveal that* Drosopohila* Atg101 shares 51% amino acid identity (111/218) and 71% similarity (156/218) with human Atg101 and 33% amino acid identity (81/244) and 46% similarity (113/244) with isoform a of worm Atg101/EPG-9, respectively (Figure 1), suggesting that it is an ortholog of Atg101 proteins. Two separate transgenic RNAi lines are available from public stock centers that allow inducible silencing of* Drosopohila Atg101*, both integrated into specific, nonrandom landing sites in the genome.* Atg101[KK] *is based on a long hairpin, while* Atg101[HMS] *contains a short, microRNA-based duplex RNA that targets a 22-nucleotide sequence in the 3′ untranslated region of the endogenous mRNA. LysoTracker Red (LTR) is widely used for the labeling of acidic autolysosomes in the larval* Drosophila *fat body, as this vital dye shows little to no punctate staining in fat body cells of well-fed larvae [9, 19, 23, 35]. Expression of either of the* Atg101* RNAi constructs in GFP-marked cell clones prevents starvation-induced LysoTracker Red- (LTR-) positive autolysosome formation (Figures 2(a), 2(b), and 2(c)). As expected, systemic expression of either of these transgenic RNAi lines strongly decreases endogenous* Atg101 *mRNA levels (Figure 2(d)). Depletion of* Atg101* also impairs the distribution of endogenous Atg8a, as instead of the numerous small Atg8a-positive autophagosomes seen in control cells, fewer but larger Atg8a structures are observed in GFP-marked* Atg101* knockdown cells (Figures 3(a), 3(b), and 3(d)). This may not be simply due to incomplete gene silencing, as these aberrant Atg8a-positive structures are also seen upon simultaneous expression of both* Atg101* RNAi constructs (Figures 3(c) and 3(d)). This phenotype is very similar to the accumulation of Atg8/LGG-1 aggregates reported in* Atg101/epg-9* mutant worms [18]. The enlarged Atg8a structures observed in* Atg101* RNAi cells of starved animals colocalize with protein aggregates containing the specific autophagy cargo ref(2)P (Figure 3(e)). Although it is not possible to conclude that Atg101 is not required for Atg8a recruitment to ref(2)P aggregates solely based on RNAi experiments, this seems to be a possibility, which is also supported by data from mutant worms. Interestingly, of the core* Atg* genes,* Atg2* has also been shown to be dispensable for Atg8a recruitment to the phagophore assembly site and protein aggregates in worm, fly, and mammalian cells [19, 24, 36, 37]. To exclude the possibility that these Atg8a dots might represent autophagosomes, we carried out electron microscopy. Ultrastructural analysis indeed revealed that autophagosome formation is blocked in* Atg101* RNAi cells (Figure 4). A potential explanation for the presence of a few enlarged Atg8a-positive dots in* Atg101* loss-of-function cells but not in* FIP200* null mutants [9] may be that mammalian Atg8 homologs have been shown to directly bind to FIP200, Atg13, and Atg1/ULKs [38]. These Atg1 kinase subunits may facilitate the recruitment of Atg8a to aggregates containing ref(2)P and ubiquitinated proteins, which have been proposed to act as scaffolds for autophagosome biogenesis, both in mammals and* Drosophila* [9, 24, 39].

### 3.2. *Drosopohila* Atg101 Is Required for Selective Basal Autophagy of Ref(2)P-Containing Protein Aggregates

**Figure 5:**
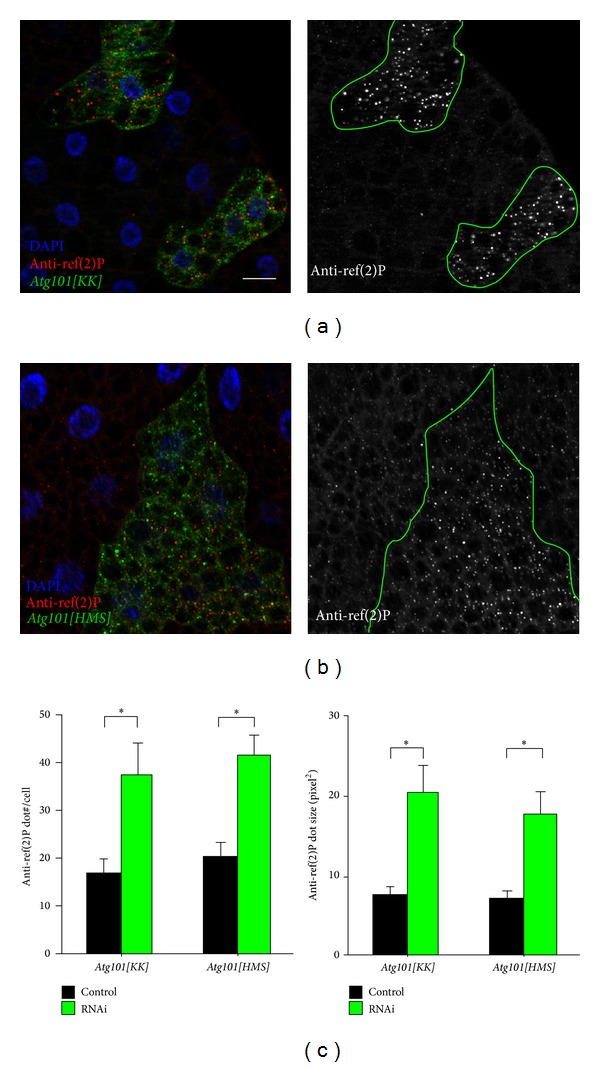
Depletion of* Atg101 *in Lamp1-GFP-marked fat body cells of well-fed larvae results in the accumulation of ref(2)P aggregates ((a), (b)). (c) Quantification of data shown in (a) and (b), *N* = 5, *P* < 0.001, two-tailed, two-sample Student's* t*-tests. Scalebar equals 20 *μ*m.

Ref(2)P appears to be selectively degraded by autophagy all the time, independent of the induction conditions such as starvation or developmental contexts, similar to its mammalian homolog p62 [24, 40]. Continuous basal autophagy is usually difficult to be visualized directly, due to its low levels. For this reason, assessing basal autophagic activity by looking at levels of ref(2)P has become a standard test, similar to mammals [14, 19, 22, 41, 42]. Expression of either of the two* Atg101 *silencing constructs results in large-scale accumulation of ref(2)P aggregates in GFP-marked cells, when compared to surrounding wild-type tissue in well-fed larvae (Figures 5(a), 5(b), and 5(c)), indicating defects in selective basal autophagy.

### 3.3. Atg101 Interacts with Atg1

**Figure 6:**
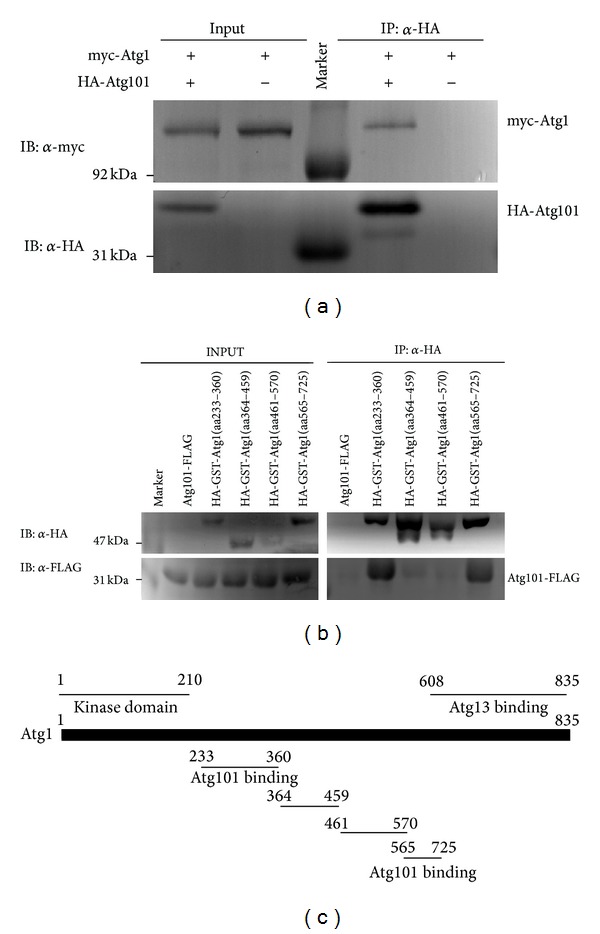
Full-length, kinase dead myc-Atg1 coprecipitates with HA-Atg101 but not with anti-HA beads (a). Atg101-FLAG coprecipitates with HA-GST-tagged Atg1 fragments 233–360 and 565–725 but not with 364–459 and 461–570 (b). Atg1 domain structure [9] and fragments used in mapping experiments (c).

The Atg13, FIP200, and ULK1 subunits of the mammalian Atg1/ULK kinase complex have been shown to bind to Atg101 in coimmunoprecipitation experiments on the level of endogenous proteins [16]. Atg101 directly binds to Atg13 in mammals, and the Atg101 ortholog EPG-9 was recently proposed to directly interact with the Atg1 ortholog UNC-51 as well in* C. elegans* [16–18]. In line with these studies, we found that myc-tagged, kinase dead Atg1 coimmunoprecipitates with Atg101 in cultured* Drosopohila* cells (Figure 6(a)). We used the kinase dead form because wild-type Atg1 is expressed poorly in cultured cells, and it also reduces the expression of other transgenes, likely due to Atg1-mediated feedback inhibition of Tor-dependent translation [9, 10, 43]. To determine which regions of the 835 amino acid long Atg1 protein are involved in its interaction with Atg101, we generated four HA-GST-tagged constructs for different Atg1 fragments. The N-terminal kinase domain was not included in these experiments, as again it is expressed poorly and strongly impairs the expression of coexpressed constructs [9]. We decided to search for potential Atg101 binding sites in the middle region (amino acids 233–725) of Atg1, because the N-terminal kinase domain is the only known domain in this protein, and the C-terminal region is involved in its binding to Atg13 [44]. The rest of the protein is predicted to be mostly unstructured by the IUPRED server, and the ANCHOR method identifies numerous potential binding sites within these unstructured regions. We found that Atg101-FLAG coimmunoprecipitates with two Atg1 fragments containing either amino acids 233–360 or 565–725 but not with fragments containing amino acids 364–459 or 461–570 (Figures 6(b) and 6(c)). These results suggest that Atg101 interacts with multiple regions in Atg1 either directly or indirectly.

### 3.4. Atg101 Binds to the HORMA Domain of Atg13, and It Can Dimerize

**Figure 7:**
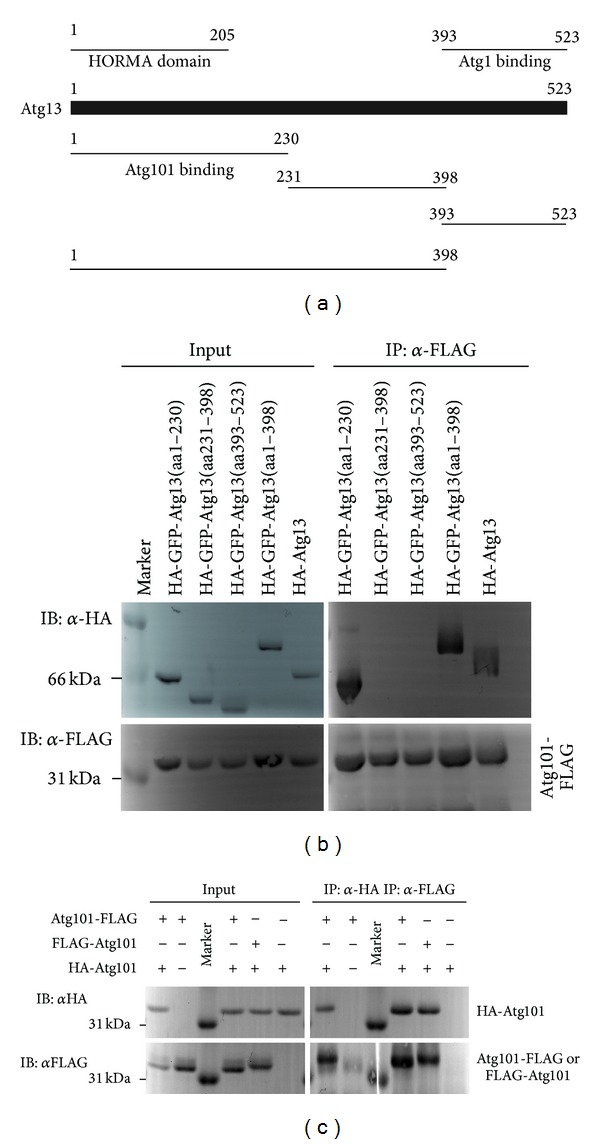
Atg13 domain structure and fragments used in mapping experiments [9] (a). Atg101-FLAG coprecipitates the full-length HA-Atg13, and also HA-GFP-Atg13 fragments containing the N-terminal HORMA domain: 1–230 and 1–398, but not the middle (231–398) or C-terminal (393–523) Atg13 fragments (b). Atg101-FLAG coprecipitates with HA-Atg101, and HA-Atg101 coprecipitates with both FLAG-Atg101 and Atg101-FLAG, but not with anti-HA or anti-FLAG beads alone, respectively (c).

Coimmunoprecipitations showed that full-length HA-Atg13 binds to Atg101-FLAG (Figure 7(b)), in line with data from mammals [16, 17]. Mapping experiments revealed that only those regions of Atg13 coprecipitate with Atg101 that contain amino acids 1–230, whereas the unstructured middle and C-terminal regions do not show binding (Figures 7(a) and 7(b)). The N-terminal part of Atg13 folds into a HORMA (Hop1, Rev1, and Mad2) domain similar to that of Mad2, a spindle checkpoint protein [45]. Mad2 forms a conformational homodimer, as its dimerization requires the binding of two different stable conformations to each other: open, O-Mad2, and closed, C-Mad2 [46]. Since the Atg13 HORMA domain is similar to C-Mad2 [45], we hypothesized that Atg101 may potentially be a HORMA domain protein as well, perhaps capable of forming either an O-Mad2 state or both O-Mad2 and C-Mad2 states. To study this model further, we first determined the interaction of Atg101 with itself. We found that Atg101-FLAG coprecipitates with HA-Atg101, and vice versa HA-Atg101 coprecipitates with Atg101 tagged by FLAG on either its N- or C-terminus (Figure 7(c)), suggesting that Atg101 dimerizes.

### 3.5. Atg101 Is Potentially a HORMA Domain Protein

**Figure 8:**
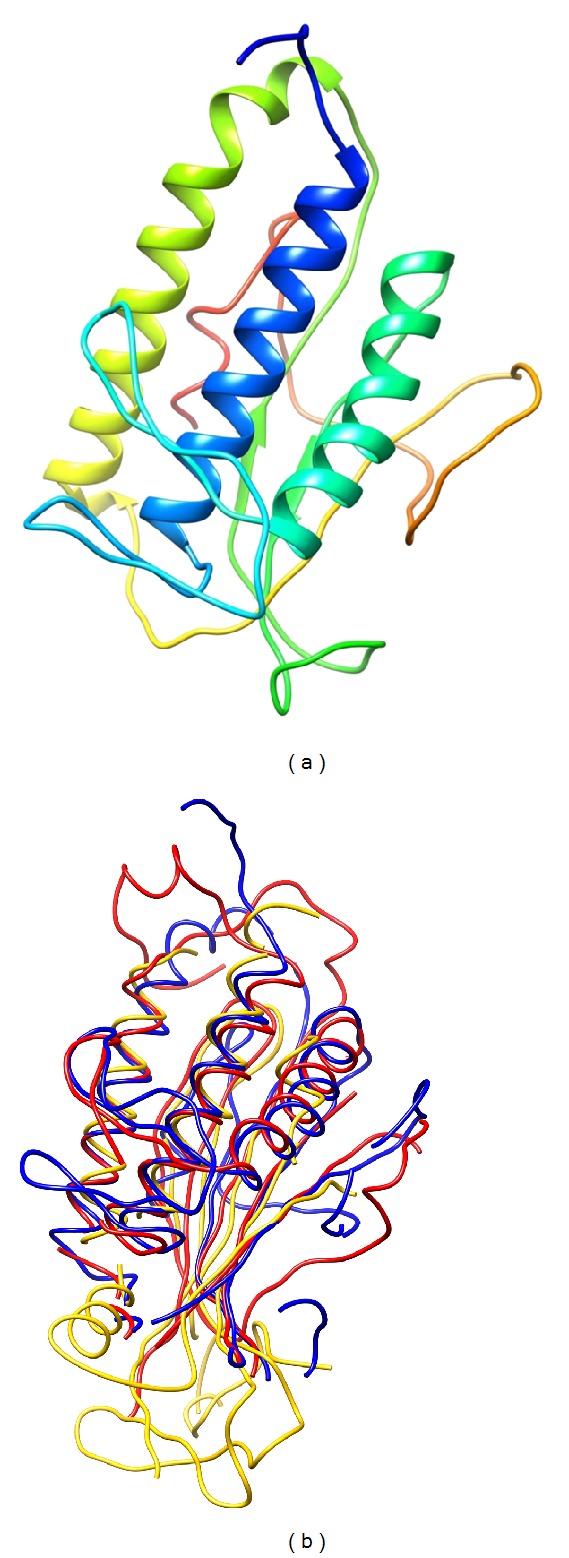
Ribbon representation of the best model of* Drosopohila* Atg101 obtained with I-TASSER (a). Atg101 structure alignment of the best model obtained with I-TASSER (blue), as well as human Mad2 (2V64, chain A, yellow) and Atg13 from the yeast* Lachancea thermotolerans* (4J2G, chain A, red) (b).

**Figure 9:**
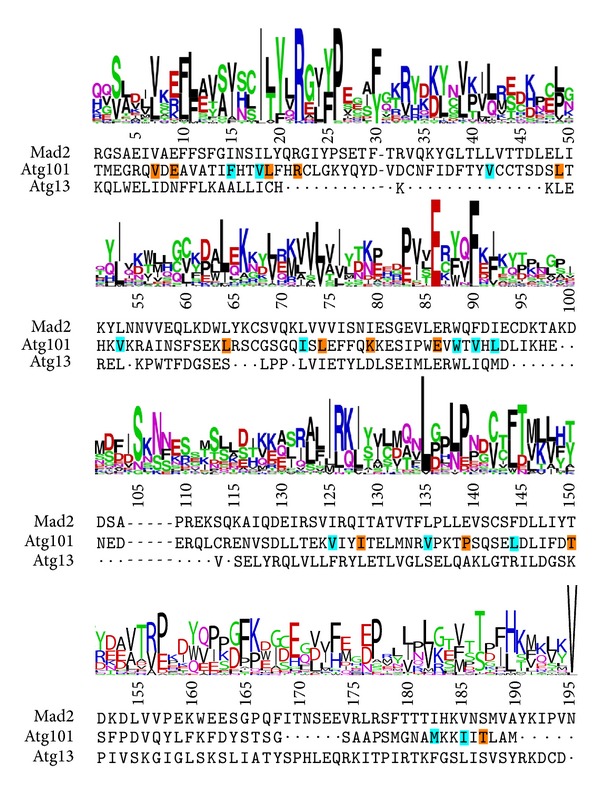
Comparison of the Prosite HORMA domain logo with the sequence alignment derived from the multiple structure alignment of the predicted Atg101 structure, Mad2 (2V64, chain A), and Atg13 (4J2G, chain A). Residues in* Drosopohila* Atg101 corresponding to the most frequent one in the Prosite logo and mostly conserved in other Atg101 proteins are highlighted with an orange background. Residues with a similar character to those in the logo and conserved in Atg101 proteins are highlighted with cyan background. Dots denote gaps arisen in the original structural alignment, dashes represent gaps relative to the Prosite logo. Note that the alignment is adjusted to match the full Prosite logo, so insertions relative to that have been removed from all sequences, and thus they do not correspond to the full native ones. In the Prosite logo, positively and negatively charged residues are colored blue and red, respectively; those with hydrophobic side chains are shown in black; Gln and Asn in magenta; other amino acids with hydrophilic side chains and glycine in green.

Atg101 is classified as a single-domain protein with a domain of unknown function (DUF1649 in Pfam), with no trivially detectable relatives of known structure. Basic structural predictions suggest that it is a structured globular cytoplasmic protein. Results obtained with I-TASSER and Phyre2 indicate that the protein might have a HORMA domain fold. Both servers identified templates with HORMA fold and three of I-TASSER's 5 predicted structures have a fold similar to each other as well as the Atg13 and Mad2 HORMA domains (Figure 8). Moreover, the model predicted by Phyre2 also has a HORMA-like structural core (not shown). To assess the plausibility of this hypothesis, we created a multiple structure alignment of the best I-TASSER model of Atg101 with the known structures of human Mad2 [46] and yeast Atg13 [45], and manually compared it to the HORMA domain profile composed of 33 proteins in Prosite (entry PS50815). Our analysis reveals a number of corresponding sites showing conservation both in the HORMA domains in Prosite and within Atg101 homologs (OMA group 413828 containing Atg101 proteins from 66 different species). In addition, a characteristic pattern of largely conserved Leu/Ile/Val residues in Atg101 proteins and matching hydrophobic residues in the Prosite profile is apparent (Figure 9).

### 3.6. Atg101 Interacts with Ref(2)P/p62

**Figure 10:**
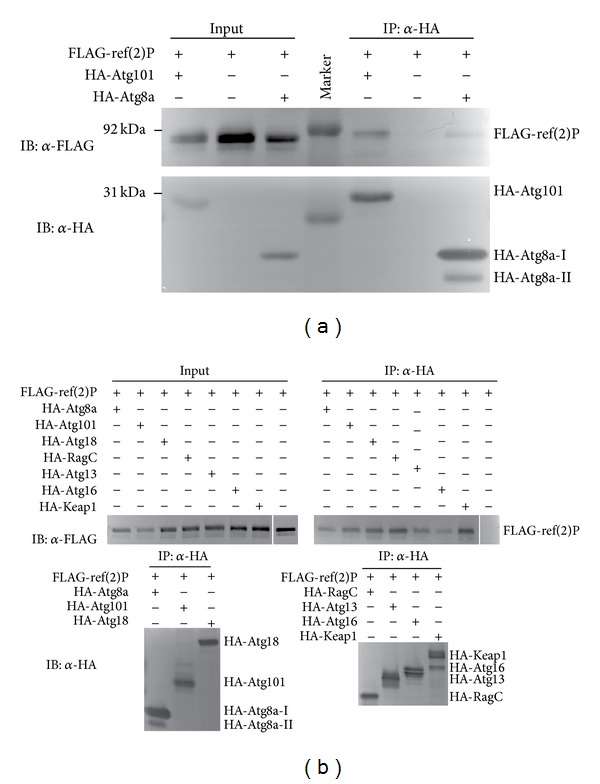
FLAG-tagged ref(2)P coprecipitates with both HA-Atg101 and HA-Atg8a, but not with anti-HA beads (a). Note that HA-Atg8a-II migrates faster than HA-Atg8a-I, due to the covalent attachment of the lipid moiety phosphatidylethanolamine to its C-terminus. (b) The selective autophagy cargo ref(2)P coprecipitates with Atg8a, Atg101, Atg18, RagC, Atg13, Atg16, and Keap1, but not with empty beads.

*Drosopohila* Atg101 was suggested to bind to ref(2)P and also to several subunits of the lysosomal proton pump v-ATPase complex (Vha26, Vha36-1, Vha44, Vha55, and Vha68-2) in a large-scale proteomic study [47]. As we have recently found that the Atg1 kinase subunit FIP200 frequently localizes to ref(2)P aggregates near lysosomes [9], we wished to confirm the proposed interaction of Atg101 with ref(2)P. Indeed, FLAG-tagged ref(2)P coprecipitated with HA-Atg101 (Figure 10(a)). We used HA-Atg8a as a positive control in this experiment, as Atg8 homologs are established binding partners of p62 in mammalian cells, and fly Atg8a has been proposed to bind to ref(2)P through a conserved Atg8 interacting region as well [14, 15, 48]. These data raised the possibility that the entire Atg1 kinase complex may bind to aggregates of ref(2)P and ubiquitinated proteins. Indeed, we found that Atg13 also shows an interaction with ref(2)P, similar to Atg101 (Figure 10(b)). Positive controls that we used in these coimmunoprecipitations include Keap1 and Atg18, both of which have recently been shown to bind to ref(2)P [20, 24]. Ref(2)P coprecipitates with additional critical regulators of autophagy as well: the ubiquitin-binding protein Atg16 [49] and the small GTPase RagC, a known Vha complex-associated regulator of Tor kinase [4], which shows a particularly strong interaction with ref(2)P as inferred from a large-scale proteomic study [47] (Figure 10(b)). These data are in line with the potential role of lysosome-associated protein aggregates as scaffolds for autophagosome biogenesis [9, 24, 39].

## 4. Conclusions

Our data establish that Atg101 is required for autophagy in* Drosopohila*, and that it is a subunit of the Atg1 kinase complex, similar to mammals and worms [16–18]. The potential binding of Atg101 to Atg1 may involve multiple regions in Atg1, whereas the HORMA domain of Atg13 appears to be the only region important for its interaction with Atg101. Atg101 is a small protein of 218 amino acids, and it is predicted to fold into a globular domain, potentially a HORMA domain. Proteins with such structure often have numerous binding partners, the identity of which determines the open or closed conformation in the case of Mad2 [46]. Our bioinformatics analysis predicts a possible HORMA domain structure for Atg101, and we show that it can indeed dimerize. If Atg101 functions similar to Mad2, then it may perhaps be capable of switching between open and closed conformations and form a conformational homodimer as well. This would represent an elegant way of determining which proteins can bind to Atg101 at one time. This model predicts that Atg101 binds to Atg13 through direct interaction of the two HORMA domains, whereas for some of its other partners (potentially including Atg1), certain oligopeptides located in unstructured regions may engage in an interaction with Atg101, as seen in the case of Mad2 as well [46].

It is important to emphasize that the HORMA domain structure of Atg101 is not supported experimentally. Further analysis of Atg101 binding partners, fine mapping of the binding sites mediating the interaction of each partner with Atg101, and analysis of the Atg101 domain by structural biochemistry will be necessary to test our hypothetical model presented in this paper. This will be particularly important, as the absence of an Atg101 ortholog in yeast clearly indicates that a metazoan-specific regulatory mechanism has evolved to control the initiation step of autophagy in animal cells.

## References

[1] Klionsky DJ, Cregg JM, Dunn WA (2003). A unified nomenclature for yeast autophagy-related genes. *Developmental Cell*.

[2] Erdi B, Nagy P, Zvara A (2012). Loss of the starvation-induced gene Rack1 leads to glycogen deficiency and impaired autophagic responses in Drosophila. *Autophagy*.

[3] Mizushima N (2010). The role of the Atg1/ULK1 complex in autophagy regulation. *Current Opinion in Cell Biology*.

[4] Zoncu R, Bar-Peled L, Efeyan A, Wang S, Sancak Y, Sabatini DM (2011). mTORC1 senses lysosomal amino acids through an inside-out mechanism that requires the vacuolar H+-ATPase. *Science*.

[5] Juhasz G (2012). Interpretation of bafilomycin, pH neutralizing or protease inhibitor treatments in autophagic flux experiments: novel considerations. *Autophagy*.

[6] Hosokawa N, Hara T, Kaizuka T (2009). Nutrient-dependent mTORCl association with the ULK1-Atg13-FIP200 complex required for autophagy. *Molecular Biology of the Cell*.

[7] Banreti A, Lukacsovich T, Csikos G, Erdelyi M, Sass M (2012). PP2A regulates autophagy in two alternative ways in Drosophila. *Autophagy*.

[8] Chang Y-Y, Neufeld TP (2009). An Atg1/Atg13 complex with multiple roles in TOR-mediated autophagy regulation. *Molecular Biology of the Cell*.

[9] Nagy P, Kárpáti M, Varga A (2014). Atg17/FIP200 localizes to perilysosomal Ref(2)P aggregates and promotes autophagy by activation of Atg1 in Drosophila. *Autophagy*.

[10] Scott RC, Juhász G, Neufeld TP (2007). Direct induction of autophagy by Atg1 inhibits cell growth and induces apoptotic cell death. *Current Biology*.

[11] Ichimura Y, Kirisako T, Takao T (2000). A ubiquitin-like system mediates protein lipidation. *Nature*.

[12] Mizushima N, Yamamoto A, Matsui M, Yoshimori T, Ohsumi Y (2004). In vivo analysis of autophagy in response to nutrient starvation using transgenic mice expressing a fluorescent autophagosome marker. *Molecular Biology of the Cell*.

[13] Johansen T, Lamark T (2011). Selective autophagy mediated by autophagic adapter proteins. *Autophagy*.

[14] Pankiv S, Clausen TH, Lamark T (2007). p62/SQSTM1 binds directly to Atg8/LC3 to facilitate degradation of ubiquitinated protein aggregates by autophagy. *Journal of Biological Chemistry*.

[15] Nezis IP, Simonsen A, Sagona AP (2008). Ref(2)P, the Drosophila melanogaster homologue of mammalian p62, is required for the formation of protein aggregates in adult brain. *Journal of Cell Biology*.

[16] Hosokawa N, Sasaki T, Iemura S-I, Natsume T, Hara T, Mizushima N (2009). Atg101, a novel mammalian autophagy protein interacting with Atg13. *Autophagy*.

[17] Mercer CA, Kaliappan A, Dennis PB (2009). A novel, human Atg13 binding protein, Atg101, interacts with ULK1 and is essential for macroautophagy. *Autophagy*.

[18] Liang Q, Yang P, Tian E, Han J, Zhang H (2012). The C. elegans ATG101 homolog EPG-9 directly interacts with EPG-1/Atg13 and is essential for autophagy. *Autophagy*.

[19] Takats S, Nagy P, Varga Á (2013). Autophagosomal syntaxin17-dependent lysosomal degradation maintains neuronal function in Drosophila. *The Journal of Cell Biology*.

[20] Nagy P, Varga A, Pircs K, Hegedus K, Juhasz G (2013). Myc-driven overgrowth requires unfolded protein response-mediated induction of autophagy and antioxidant responses in drosophila melanogaster. *PLOS Genetics*.

[21] Juhász G, Érdi B, Sass M, Neufeld TP (2007). Atg7-dependent autophagy promotes neuronal health, stress tolerance, and longevity but is dispensable for metamorphosis in Drosophila. *Genes and Development*.

[22] Pircs K, Nagy P, Varga A (2012). Advantages and limitations of different p62-based assays for estimating autophagic activity in Drosophila. *PloS ONE*.

[23] Juhasz G, Neufeld TP (2008). Experimental control and characterization of autophagy in drosophila. *Methods in Molecular Biology*.

[24] Nagy P, Hegedus K, Pircs K, Varga A, Juhasz G (2014). Different effects of Atg2 and Atg18 mutations on Atg8a and Atg9 trafficking during starvation in Drosophila. *FEBS Letters*.

[25] Altenhoff AM, Schneider A, Gonnet GH, Dessimoz C (2011). OMA 2011: orthology inference among 1000 complete genomes. *Nucleic Acids Research*.

[26] Roy A, Kucukural A, Zhang Y (2010). I-TASSER: a unified platform for automated protein structure and function prediction. *Nature Protocols*.

[27] Kelley LA, Sternberg MJE (2009). Protein structure prediction on the Web: a case study using the Phyre server. *Nature Protocols*.

[28] Holm L, Park J (2000). DaliLite workbench for protein structure comparison. *Bioinformatics*.

[29] Lupyan D, Leo-Macias A, Ortiz AR (2005). A new progressive-iterative algorithm for multiple structure alignment. *Bioinformatics*.

[30] Meszaros B, Dosztanyi Z, Simon I (2012). Disordered binding regions and linear motifs—bridging the gap between two models of molecular recognition. *PloS ONE*.

[31] Dosztányi Z, Csizmok V, Tompa P, Simon I (2005). IUPred: web server for the prediction of intrinsically unstructured regions of proteins based on estimated energy content. *Bioinformatics*.

[32] Pettersen EF, Goddard TD, Huang CC (2004). UCSF Chimera—a visualization system for exploratory research and analysis. *Journal of Computational Chemistry*.

[33] Crooks GE, Hon G, Chandonia J-M, Brenner SE (2004). WebLogo: a sequence logo generator. *Genome Research*.

[34] Subramaniam S (1998). The Biology Workbench—a seamless database and analysis environment for the biologist. *Proteins*.

[35] Scott RC, Schuldiner O, Neufeld TP (2004). Role and regulation of starvation-induced autophagy in the Drosophila fat body. *Developmental Cell*.

[36] Lu Q, Yang P, Huang X (2011). The WD40 repeat ptdIns(3)P-binding protein EPG-6 regulates progression of omegasomes to autophagosomes. *Developmental Cell*.

[37] Velikkakath AKG, Nishimura T, Oita E, Ishihara N, Mizushima N (2012). Mammalian Atg2 proteins are essential for autophagosome formation and important for regulation of size and distribution of lipid droplets. *Molecular Biology of the Cell*.

[38] Alemu EA, Lamark T, Torgersen KM (2012). ATG8 family proteins act as scaffolds for assembly of the ULK complex: sequence requirements for LC3-interacting region (LIR) motifs. *The Journal of Biological Chemistry*.

[39] Rogov V, Dotsch V, Johansen T, Kirkin V (2014). Interactions between autophagy receptors and ubiquitin-like proteins form the molecular basis for selective autophagy. *Molecular Cell*.

[40] Komatsu M, Waguri S, Koike M (2007). Homeostatic levels of p62 control cytoplasmic inclusion body formation in autophagy-deficient mice. *Cell*.

[41] Bartlett BJ, Isakson P, Lewerenz J (2011). p62, Ref(2)P and ubiquitinated proteins are conserved markers of neuronal aging, aggregate formation and progressive autophagic defects. *Autophagy*.

[42] Klionsky DJ, Abdalla FC, Abeliovich H (2012). Guidelines for the use and interpretation of assays for monitoring autophagy. *Autophagy*.

[43] Jung CH, Seo M, Otto NM, Kim D-H (2011). ULK1 inhibits the kinase activity of mTORC1 and cell proliferation. *Autophagy*.

[44] Jung CH, Jun CB, Ro S-H (2009). ULK-Atg13-FIP200 complexes mediate mTOR signaling to the autophagy machinery. *Molecular Biology of the Cell*.

[45] Jao CC, Ragusa MJ, Stanley RE, Hurley JH (2013). A HORMA domain in Atg13 mediates PI 3-kinase recruitment in autophagy. *Proceedings of the National Academy of Sciences of the United States of America*.

[46] Mapelli M, Massimiliano L, Santaguida S, Musacchio A (2007). The Mad2 conformational dimer: structure and implications for the spindle assembly checkpoint. *Cell*.

[47] Guruharsha KG, Rual J-F, Zhai B (2011). A protein complex network of Drosophila melanogaster. *Cell*.

[48] Nezis IP (2012). Selective autophagy in Drosophila. *International Journal of Cell Biology*.

[49] Fujita N, Morita E, Itoh T (2013). Recruitment of the autophagic machinery to endosomes during infection is mediated by ubiquitin. *The Journal of Cell Biology*.

